# Numerical Modeling of Gentamicin Transport in Agricultural Soils: Implications for Environmental Pollution

**DOI:** 10.3390/antibiotics14080786

**Published:** 2025-08-02

**Authors:** Nami Morales-Durán, Sebastián Fuentes, Jesús García-Gallego, José Treviño-Reséndez, Josué D. García-Espinoza, Rubén Morones-Ramírez, Carlos Chávez

**Affiliations:** 1 Chemical Sciences Faculty, Autonomous University of Nuevo Leon, San Nicolas de los Garza 66451, Mexico; nami.moralesd@uanl.edu.mx; 2 Research Center for Biotechnology and Nanotechnology, Chemical Sciences Faculty, Research and Technological Innovation Park, Autonomous University of Nuevo Leon, Apodaca 66629, Mexico; 3 Water Research Center, Department of Irrigation and Drainage Engineering, Autonomous University of Queretaro, Cerro de las Campanas SN, Col. Las Campanas, Santiago de Querétaro 76010, Mexico; sebastian.fuentes@uaq.mx (S.F.); jgarcia341@alumnos.uaq.mx (J.G.-G.); 4 Centro de Investigación en Química para la Economía Circular—CIQEC, Facultad de Química, Universidad Autónoma de Querétaro, Cerro de las Campanas SN, Col. Las Campanas, Santiago de Querétaro 76010, Mexico; jose.trevino@uaq.edu.mx (J.T.-R.); josue.daniel.garcia@uaq.mx (J.D.G.-E.)

**Keywords:** antibiotic pollution, farm soil, advection dispersion equation, Richards equation, inverse modelling

## Abstract

**Background/Objectives:** In recent years, the discharge of antibiotics into rivers and irrigation canals has increased. However, few studies have addressed the impact of these compounds on agricultural fields that use such water to meet crop demands. **Methods:** In this study, the transport of two types of gentamicin (pure gentamicin and gentamicin sulfate) was modeled at concentrations of 150 and 300 μL/L, respectively, in a soil with more than 60 years of agricultural use. Infiltration tests under constant head conditions and gentamicin transport experiments were conducted in acrylic columns measuring 14 cm in length and 12.7 cm in diameter. The scaling parameters for the Richards equation were obtained from experimental data, while those for the advection–dispersion equation were estimated using inverse methods through a nonlinear optimization algorithm. In addition, a fractal-based model for saturated hydraulic conductivity was employed. **Results:** It was found that the dispersivity of gentamicin sulfate is 3.1 times higher than that of pure gentamicin. Based on the estimated parameters, two simulation scenarios were conducted: continuous application of gentamicin and soil flushing after antibiotic discharge. The results show that the transport velocity of gentamicin sulfate in the soil may have short-term consequences for the emergence of resistant microorganisms due to the destination of wastewater containing antibiotic residues. **Conclusions:** Finally, further research is needed to evaluate the impact of antibiotics on soil physical properties, as well as their effects on irrigated crops, animals that consume such water, and the soil microbiota.

## 1. Introduction

The use of antibiotics for treating infections in both animals and humans has increased substantially in recent years. By 2030, an estimated 105,596 (±3605) tons of antibiotics will be required for animal feed due to the rising demand for livestock raised for food production [1]. These compounds, originating from agricultural, industrial, and urban sources, may leach into water bodies, affect soil microbiota, and re-enter the food chain through accumulation in plants and animals [2,3,4].

Soil microorganisms represent the most abundant and diverse biological communities in the natural environment. It has been documented that a single gram of soil harbors a wide range of microbial diversity, including bacteria, fungi, viruses, archaea, algae, and protozoa [5,6,7]. Among them, bacteria and fungi stand out as the most relevant groups within the soil ecosystem [8].

Aminoglycosides, such as gentamicin, are bactericidal antibiotics widely used across human, animal, and plant health sectors [9,10,11]. They are poorly absorbed in the gastrointestinal tract and are excreted largely unmetabolized, promoting the emergence of antibiotic resistance through their interaction with microorganisms in the environment [12].

Gentamicin is an aminoglycoside widely used in both human and veterinary medicine, and high concentrations of this antibiotic have been reported in wastewater treatment plants and in soil [13,14]. It has also been shown that gentamicin is readily absorbed by commercially important plants [15]. This antibiotic has also been used in the treatment of late blight, a disease caused by the protist *Phytophthora infestans*, which affects apples and pears. In addition, it has been applied to combat various diseases in certain vegetables grown in Mexico and in some regions of South America [16]. Although resistance to gentamicin has been widely documented in bacteria affecting humans and animals, there is limited information regarding its prevalence in plant-associated bacteria, as well as the behavior of the antibiotic in soil [10,16].

Aminoglycoside antibiotics are among the most commonly prescribed antibiotics worldwide due to their antimicrobial efficacy, wide availability, and low cost [17]. Gentamicin is a prescription aminoglycoside approved by the United States Food and Drug Administration (FDA) for the treatment of various bacterial infections. It is used in cases such as meningitis, septicemia, and severe urinary tract infections, and is also effective against certain opportunistic bacterial infections [18]. Gentamicin sulfate, on the other hand, is a water-soluble salt that corresponds to a broad-spectrum aminoglycoside antibiotic complex produced by the fermentation of *Micromonospora purpurea* or *Micromonospora echinospora* with demonstrated antibacterial activity [18].

Mathematical models enhance our understanding of antibiotic transport in soil and groundwater, particularly when supported by laboratory experiments [19]. Process-based models, which combine theoretical principles with empirical observations, are especially useful for simulating the fate of antibiotics in soil [20].

Among mathematical models, the advection–dispersion equation [21] has been widely used to describe solute transport in porous media such as soil [22]. This partial differential equation represents the combined effects of advection, the transport of solutes driven by the flow of the medium, and dispersion, which describes solute diffusion resulting from velocity gradients within the fluid. Additionally, the Richards equation addresses water movement in the porous medium [23], which supports the simulation of contaminant transport.

Recent studies have proposed analytical solutions to the one-dimensional advection and dispersion equation in contaminated soils, considering different boundary conditions such as first-type (Dirichlet) [24] or third-type (Robin) [24,25]. These boundary conditions provide an accurate description of mass conservation but may be difficult to implement in computational tools that use different boundary formulations. Moreover, extrapolations based on Dirichlet boundary conditions may underestimate concentration profiles. These factors highlight the importance of carefully selecting boundary conditions and considering the inherent limitations of these solutions in practical applications.

It is also important to consider other factors such as climatic conditions, which can modify or alter the physical properties of the soil [26], as well as the spatial variability of soil that may exist among different agricultural plots [27].

Mathematical models are essential for representing and predicting subsurface dynamics. The Richards equation [28] and the advection–dispersion equation are particularly robust modeling tools. Their strength lies in their ability to describe complex physical phenomena and their versatility to adapt to a wide range of boundary conditions. This flexibility allows their application in various scenarios and ensures accurate and reliable results. This characteristic robustness has made them the foundation for studying water and solute transport in the subsurface [29,30].

Recent studies have confirmed that the coupling of the Richards equation with the advection–dispersion equation remains one of the most effective frameworks for simulating water and solute movement in variably saturated soils. This approach offers flexibility to calibrate key hydraulic and transport parameters—such as K_s_, $\Psi_{d}$, and dispersion coefficients—that are often difficult to measure directly in the field. Moreover, it allows adaptation to heterogeneous soil textures and layered profiles, which are common in real agricultural scenarios. Applications of this model structure have proven successful across a variety of contexts, from metal leaching in mine tailings [31] to nitrogen transport enhanced with physics-informed neural networks [32,33], and fungicide transport in agricultural soils using coupled physicochemical models [34]. These findings support the use of the Richards–ADE framework as a robust and scalable solution for evaluating contaminant transport in soil.

The objective of this study is to model the transport of gentamicin in agricultural soils using the advection–dispersion equation coupled with the Richards equation, assuming that gentamicin is primarily concentrated in the liquid phase.

## 2. Results and Discussion

### 2.1. Retention Curve and Granulometric Curve Parameters

**Figure 1:**
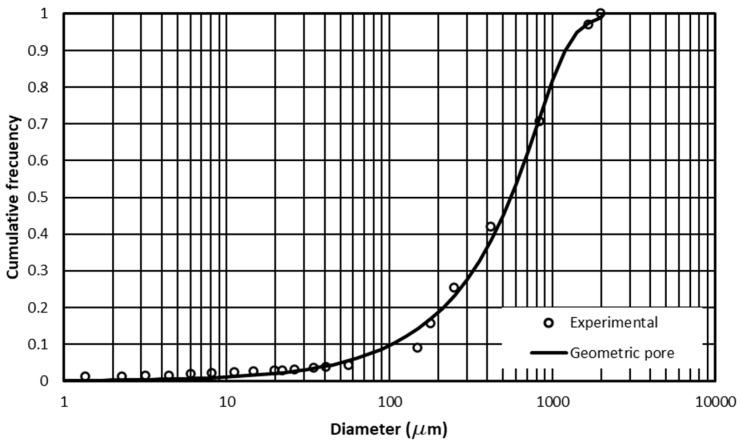
Experimental and fitted grain-size distribution curves. The circles represent the experimental cumulative particle size distribution and the solid line corresponds to the fitted curve obtained using the geometric pore model.

Shape parameters (m and n) of the water retention curve, Equation (4), were obtained from the granulometric curve (Figure 1) by fitting it with the equation $F\left(D\right)={\left[1+{\left(D_{d}/D\right)}^{\left(n/2\left(1-s\right)\right)}\right]}^{-m}$ [35], where F(D) is the cumulative frequency whose diameters are smaller than D and $D_{d}$ is a soil particle size characteristic parameter. The value of bulk density ($\rho_{a}$) was used to estimate soil porosity using the relation $\phi =1-\left(\rho_{a}/\rho_{s}\right)$, where $\rho_{s}$ is the value of the density of the quartz particles, taken as 2.65 g/cm^3^. Finally, Equation (6) was used to calculate the relative fractal dimension using the Newton–Raphson root search method. Table 1 shows the values obtained for the soils analyzed.

### 2.2. Infiltration Tests

Two steady-state infiltration tests (case 1 and case 2) were conducted with a constant water head maintained on the soil surface (h = 3.5 cm). The Richards equation was solved using a finite difference scheme following the methodology proposed by Fuentes et al. [23], using as input data the parameters of the soil water retention curve, the column length, bulk density, water depth on the soil surface, time, and cumulative infiltrated depth. The scaling parameters ($\Psi_{d}$, $K_{s}$) of Equations (4) and (5) were calculated by an inverse process using a nonlinear optimization algorithm [36]. The value of the moisture content at saturation was assumed to be equal to the porosity ($\theta_{s}=\phi$) [37] and the residual moisture content was set to zero $\left(\theta_{r}=0\right)$ [38]. Constant spatiotemporal increments were used throughout the entire calculation process (Δz= 0.001 cm and Δt= 5 × 10^−6^ h). The results of the simulation process are shown in Table 1.

### 2.3. The Transport Process and the Chemical Parameters of the Soil

The same numerical scheme used to solve the water transfer equation was used to solve the transport equation [23]; however, it is assumed that the antibiotic is mainly concentrated in the liquid phase. The water flux (q) and moisture content for all times (θ) are taken from the water transfer equation and included in the solution. For the experiments, an initial concentration of antibiotic of $C_{0}=150\;\mu L/L$ was adopted, using pure gentamicin for case 1, while for case 2 gentamicin sulfate was used at a concentration of $C_{0}=300\;\mu L/L$. Table 2 shows the results of the chemical parameters obtained in the soil sample.

**Figure 2:**
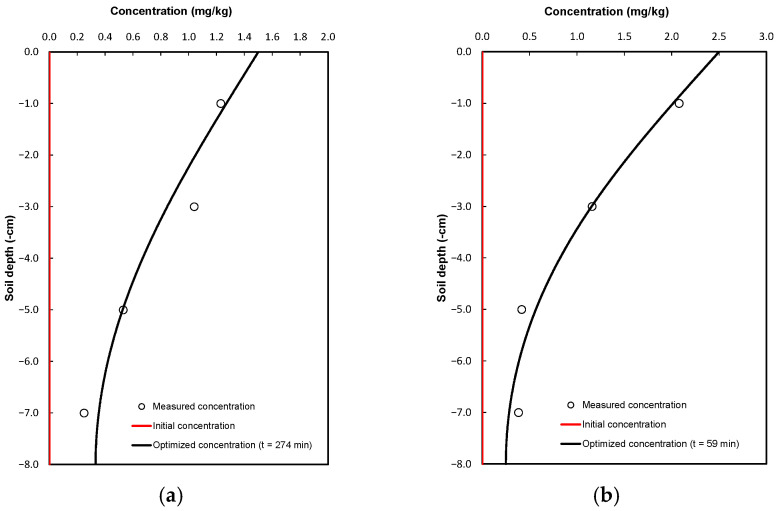

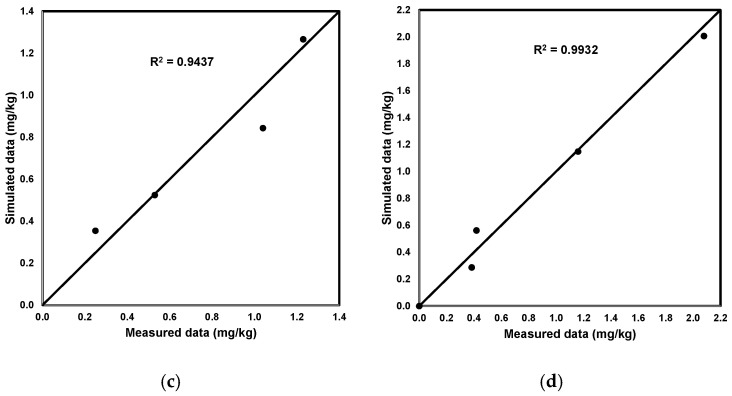
Experimental and simulated gentamicin concentration profiles in soil columns: (**a**) pure gentamicin at 274 min, and (**b**) gentamicin sulfate at 59 min. Circles represent measured values; lines represent model outputs after optimization. Comparison between measured and simulated values for gentamicin concentration: (**c**) pure gentamicin and (**d**) gentamicin sulfate.

After completing the infiltration tests, each soil column was sectioned every two centimeters, and the samples were analyzed in the laboratory to determine the solute concentration in the soil. In this case, samples were taken at depths of 1, 3, 5, and 7 cm. The results are presented in Figure 2.

The dispersion coefficient values obtained after the optimization process are presented in Table 3, where it can be observed that the dispersion coefficient of gentamicin sulfate is 3.1 times higher than that of pure gentamicin. The experimental values were measured in the laboratory at the end of the infiltration test, that is, when the soil column reached saturation. In this case, saturation was reached at 274 min when pure gentamicin was applied, and at 59 min when commercial gentamicin was used. This difference is mainly attributed to the fact that commercial gentamicin is formulated to dissolve rapidly in the bloodstream. This observation is consistent with the saturated hydraulic conductivity values shown in Table 1, where the conductivity is lower when pure gentamicin is applied ($K_{s}=$ 0.9481 cm/h) and higher when gentamicin sulfate is applied ($K_{s}=$ 1.468 cm/h).

In addition to the concentration profiles by depth, comparison graphs were created between the experimentally measured gentamicin concentrations and those simulated by the model for each formulation. These graphs allow for a visual assessment of the quality of the fit obtained through the inverse optimization process. As a complement to the RMSE, the coefficient of determination R^2^ was calculated to quantify the proportion of observed variability explained by the model. The R^2^ values obtained indicate a satisfactory fit in both cases, supporting the validity of the numerical approach employed. The results are presented in Figure 2c,d, where good agreement is observed between the model predictions and the experimental data.

### 2.4. Simulation Scenarios

**Figure 3:**
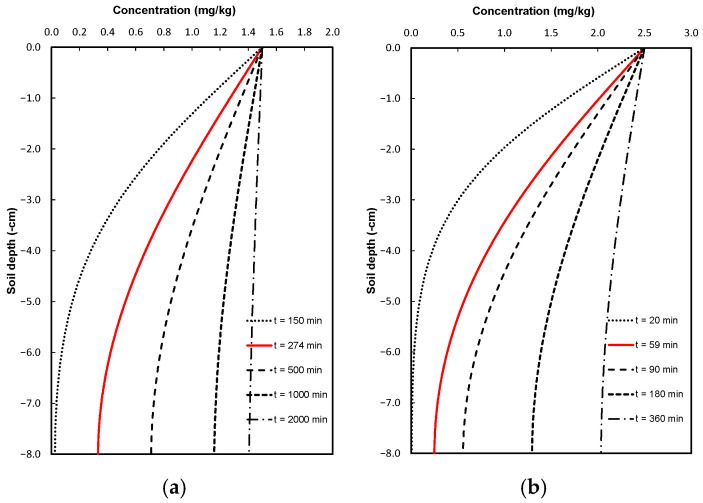
Simulated gentamicin concentration profiles at different application times: (**a**) pure gentamicin and (**b**) gentamicin sulfate.

**Figure 4:**
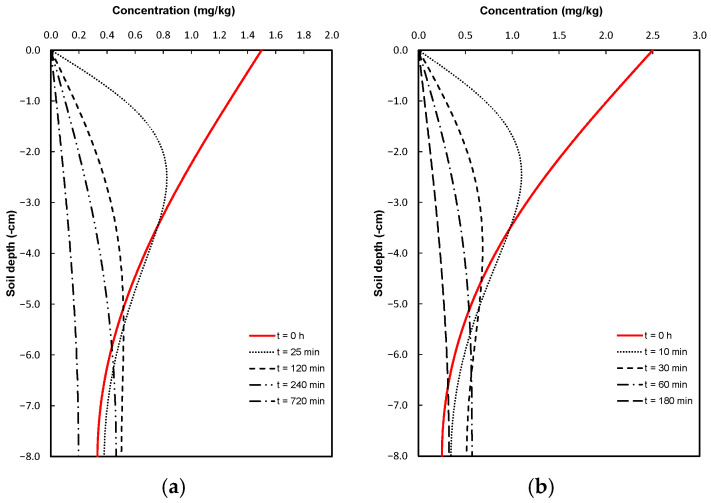
Simulated soil flushing scenarios: (**a**) pure gentamicin and (**b**) gentamicin sulfate. Curves represent solute removal over time after the end of application (t = 0 h).

Once the dispersivity of pure gentamicin and gentamicin sulfate was optimized using the laboratory-measured values, two possible scenarios were proposed. The first scenario involved continuing the application of the same gentamicin concentration over a longer period: t = 2000 min for the pure gentamicin case and t = 360 min for gentamicin sulfate (Figure 3). In the second scenario, a soil flushing process was simulated. After the soil reached saturation, infiltration continued with the application of pure water. In this case, water was added for 720 min in the pure gentamicin experiment and for 180 min in the gentamicin sulfate experiment (Figure 4).

In Scenario 1, gentamicin concentrations increased significantly within the soil profile. The soil proved to be more vulnerable under continued application of commercial gentamicin, as it moves at a velocity 5.5 times greater than that of pure gentamicin. This results in the contamination of deeper soil layers over a longer period, and in the near future, such concentrations could potentially impact groundwater sources. However, although the transport velocity of pure gentamicin is lower, the risk remains significant, but with a longer timeframe to implement soil remediation strategies to prevent further contamination.

For Scenario 2 (soil flushing), time zero represents the concentrations obtained at the moment gentamicin application was stopped at the top of the column, and clean water application began. Although Figure 4 shows that the behavior is similar to that of the previous case, the soil flushing rate is four times higher for gentamicin sulfate compared to pure gentamicin.

## 3. Discussion

The growing concern over antimicrobial resistance (AMR) has highlighted the urgent need to understand the multiple factors contributing to its spread in the environment [39]. Bacteria are in constant interaction with resources used by humans and animals, and antibiotics play a central role in these dynamics. Their intensive use, both in disease treatment and animal production, has created selective pressure that promotes the emergence and dissemination of resistant strains [40,41]. One of the main drivers of this issue is the inadequate management of waste, particularly the insufficient treatment of wastewater [40].

The inability to completely remove antibiotics and bacteria with the potential to acquire AMR has facilitated their persistence in various environments, including soil [42,43,44]. This medium, considered the most important biological resource globally, supports essential processes for human and animal life, such as the growth of food crops [45]. However, it also acts as a reservoir for pathogenic microorganisms and resistance genes, making it a critical focal point for AMR research [42]. The ability of bacteria to exchange genetic material through mechanisms such as conjugation, transformation, and transduction accelerates the spread of antimicrobial resistance genes (ARGs), turning it into a global threat [46,47].

Numerous studies have shown that the presence of antibiotics in soil, especially at high concentrations, can exert selective pressure that favors the growth of resistant bacteria, altering the antimicrobial sensitivity of entire microbial communities [12,48,49]. This phenomenon is not limited to high concentrations: even subinhibitory levels—below the minimum inhibitory concentration (MIC)—can induce genetic changes in bacterial genomes and facilitate the horizontal transfer of ARGs, along with mobile genetic elements (MGEs) such as plasmids, transposons, and genomic islands [12,50,51,52,53]. These genetic transfer mechanisms represent a critical pathway for the dissemination of AMR, as they enable the spread of ARGs even among phylogenetically distant bacterial species, expanding the ecological and public health implications of the problem [54]. Moreover, indigenous soil bacteria can act as environmental reservoirs of resistance genes, which have the potential to be transferred to bacteria that colonize the human body, posing direct risks to public health [12,55]. These findings underscore the need to adopt integrative approaches that consider both the physicochemical dynamics of antibiotic transport and the microbiological and genetic processes that determine their environmental impact. In this regard, mathematical models applied to the study of antibiotic transport in soils must evolve to incorporate relevant microbiological variables, such as ARG abundance and MGE presence, in order to enhance their predictive capacity and usefulness in risk assessment. Integrating genomic and microbiological data into simulation models would enable a more comprehensive understanding of the processes underlying AMR persistence and dissemination in agricultural ecosystems.

Gentamicin sulfate is a widely used drug obtained through the isolation and purification of a gentamicin complex composed of aminoglycoside antibiotics produced by certain microorganisms. The main components of this complex are the group C gentamicins, particularly gentamicins C1, C1a, and C2 [56]. These congeners exhibit subtle differences in chemical structure as well as in their ionic and hydrophilic behavior, which influence their mobility in aqueous environments [57,58].

It has been demonstrated that gentamicin sulfate is more water soluble than analytical-grade gentamicin. The technical data sheet of the gentamicin sulfate used indicates a solubility of up to 80 mg/mL, whereas gentamicin base exhibits a much lower solubility (approximately 50 mg/mL) [59]. This difference is attributed to the ionic charge of the sulfate, which enables effective interactions with water molecules, thereby enhancing its dissolution [60].

In contrast, analytical-grade gentamicin lacks these ionic charges, which hinders its solubilization; in some cases, it may even be insoluble in alcohol and other organic solvents. For this reason, gentamicin is almost always used in its dissolved sulfate form for pharmaceutical and laboratory applications, making it ideal for injectable formulations, eye drops, or culture media [61].

In this context, gentamicin sulfate, being a highly water-soluble salt, exhibits greater ease of dissolution and, consequently, enhanced availability for transport with water flow in soil [62]. Analytical-grade gentamicin, however, due to its markedly lower solubility, displays inherent limitations in its capacity to disperse within this medium. As a result, the elevated solubility of the sulfate form confers a more efficient transport profile in aqueous soil solutions [11,63].

In addition, analytical-grade gentamicin shows a greater tendency to interact with soil particles due to its lower net charge and relatively higher hydrophobic affinity, which results in increased adsorption to soil colloids and organic matter, thereby slowing its movement [57,64]. Conversely, the sulfate form, owing to its dissociated ionic nature (gentamicin cations and sulfate anions), tends to remain in the liquid phase and exhibits reduced affinity for soil particles, thus promoting faster transport [65].

This behavior is closely linked to the influence of soil pH, as the sulfate form, upon complete dissolution, generates a mixture of cationic species that remain stable under neutral or slightly acidic pH conditions. Under such conditions, soil particles with physicochemical activity do not strongly retain these cations. In contrast, analytical-grade gentamicin may exist in neutral or partially protonated forms, which tend to be more strongly retained in soils with negatively charged surfaces [66,67].

These differences in solubility and soil interaction are further influenced by the pharmaceutical behavior and formulation characteristics of each compound. The pharmaceutical behavior and stability of both compounds can significantly influence their fate in the soil. Gentamicin sulfate, as a salt form, exhibits greater stability under varying temperature and pH conditions, which makes it the preferred choice for injectable and topical formulations [61]. In contrast, analytical-grade gentamicin base is more susceptible to degradation or precipitation outside of tightly controlled laboratory conditions, restricting its use to highly standardized experimental settings [68]. This distinction is critical in pharmaceutical development, where active ingredient stability is essential to ensure therapeutic efficacy throughout the product’s shelf life.

A key aspect of commercial formulations such as gentamicin sulfate is the presence of adjuvants and excipients, which are not limited to stabilizing the active compound but can also modulate its release, bioavailability, and safety profile [65]. Preservatives, stabilizers, pH adjusters, and isotonic agents are incorporated to ensure biological compatibility and extend product stability. However, these adjuvants may alter the physicochemical behavior of the antibiotic, affecting properties such as dissolution rate, adsorption to biological surfaces, and tissue distribution. For instance, formulations containing surfactant-type excipients may modify how gentamicin sulfate interacts with cell membranes, either enhancing or reducing its therapeutic effect [62].

From an environmental and toxicological standpoint, the chemical form of gentamicin plays a crucial role in determining its post-release behavior. Analytical-grade gentamicin tends to adsorb more strongly to solid matrices and soil particles, limiting its mobility. In contrast, gentamicin sulfate is readily soluble in water, increasing its availability and potential transport in aqueous environments [64,66]. This distinction has direct implications for the persistence of the antibiotic in aquatic and agricultural systems, and for the potential dissemination of antibiotic resistance genes associated with the improper use or disposal of pharmaceutical products containing gentamicin sulfate [63].

It is likely that the nature of gentamicin sulfate played a determining role in its higher dispersion coefficient compared to pure gentamicin (3.1 times greater). The results showed that both the transport velocity and the time required to reach saturation were lower in the case of gentamicin sulfate than in pure gentamicin.

Additionally, the coupled model employed in this study (Richards advection–dispersion) proved to be a robust tool for simulating gentamicin transport under controlled laboratory conditions. The numerical implementation, based on finite difference methods along with inverse parameter fitting using nonlinear optimization algorithms, allowed accurate reproduction of the experimental concentration profiles for both types of gentamicin. One of the main advantages of the model is its ability to incorporate soil hydraulic variability (using a fractal model for saturated hydraulic conductivity) and its adaptability to different management scenarios (such as continuous antibiotic application or post-application soil flushing). This flexibility largely stems from the model’s capacity to modify both the initial and boundary conditions of the flow and transport equations.

In the case of water flow (Richards equation), it is possible to impose first-type (known pressure), second-type (known flux), or third-type (mixed) boundary conditions, enabling the simulation of conditions such as saturated soils in contact with ponded water, rainfall recharge, or evaporation from the surface. Likewise, initial conditions can be configured to represent pre-existing moisture profiles or dry soils, allowing simulation of infiltration pulses or intermittent irrigation events.

For solute transport (advection–dispersion equation), the model allows for Dirichlet (prescribed concentration), Neumann (prescribed solute flux), or Robin (third-type) boundary conditions, which are especially useful for simulating scenarios such as contaminant sources with fixed concentrations, temporary pollutant releases, or flushing processes with concentration gradients. Initial conditions can be set as uniform (uncontaminated) or based on complex concentration profiles resulting from previous contamination events. This modular structure allows for representation of a wide variety of agricultural or industrial contexts, adapting the mathematical formulation to site-specific conditions and contaminant input types.

Finally, the model allows for clear separation of the effects of solubility, adsorption, and compound mobility. However, it also presents limitations. The need for detailed information on soil properties, as well as its high sensitivity to input parameters, may hinder its direct application in field conditions without proper calibration. Furthermore, the model does not account for microbial interactions or the biotransformation of the antibiotic, which may be relevant in large-scale studies or in biologically active soils. Despite these limitations, we consider the approach used to be a solid foundation for future research and a significant step forward in representing the environmental fate of antibiotics in agricultural soils.

## 4. Materials and Methods

### 4.1. Transference Model

The modeling of water transfer in soil has been approached through the application of the Richards equation, which has been used in various investigations that have employed techniques such as finite differences [23], centered differences [69], and the finite element [70], among others. In its one-dimensional form, it is represented by the following notation [23]:

(1) $$C\left(\Psi \right)\frac{\partial \Psi }{\partial t}=\frac{\partial }{\partial z}\left[K\left(\Psi \right)\left(\frac{\partial \Psi }{\partial z}-1\right)\right]-ϒ$$

which is the result of the combination of Darcy’s law [71]:

(2) $$q=K\left(\Psi \right)-K\left(\Psi \right)\frac{\partial \Psi }{\partial z}$$

and the continuity equation:

(3) $$\frac{\partial \theta }{\partial t}+\frac{\partial q}{\partial z}=-ϒ$$

where $C\left(\Psi \right)$ is the specific capacity, defined as the slope of the water retention curve; Ψ the water pressure in the soil; K the hydraulic conductivity of the partially saturated soil; t the time, z the spatial direction associated with the soil depth; and ϒ a sink term.

To numerically solve the Richards equation in this study, an implicit finite difference scheme adapted to the one-dimensional case was implemented. This approach is based on the Laasonen method, which ensures numerical stability under appropriate meshing conditions. The scheme used has been previously validated by [23], who demonstrated its ability to simulate infiltration, redistribution, evaporation, and percolation processes in agricultural soils under gravity irrigation conditions. In that study, the numerical solution obtained through this scheme was compared with an available analytical solution for a specific case, showing excellent agreement and confirming its accuracy.

Since the advection–dispersion equation used to model gentamicin transport has an analogous mathematical structure (a second-order partial differential equation), the same numerical scheme was employed for its solution. This ensures consistency in the treatment of boundary conditions and time increments and maintains computational stability within the coupled system.

To numerically or analytically find the solution of Equation (1), it is necessary to know the hydrodynamic characteristics of the soil, which are models that relate the hydraulic conductivity $\left(K\right)$ and pressure potential $\left(\Psi \right)$ as functions of the volumetric water content $\left(\theta \right)$.

In this work, the van Genuchten model was used to represent the water retention curve [72]:

(4) $$\Theta \left(\Psi \right)={[1+{\left(\Psi /\Psi_{d}\right)}^{n}]}^{-m};\;m\geq 0;\;n\geq 0$$

and for the hydraulic conductivity curve, the geometric mean pore model of fractal nature was used [73]:

(5) $$K\left(\Theta \right)=K_{s}{[1-{\left(1-\Theta^{1/m}\right)}^{sm}]}^{2};\;0<sm=1-2s/n<1$$

where $\Theta \left(\Psi \right)$ is the effective soil saturation defined as $[\theta \left(\Psi \right)-\theta_{r}]/\left(\theta_{s}-\theta_{r}\right)$; $\theta_{r}$ the residual moisture content, $\theta_{r}$ the moisture content at saturation; $\Psi_{d}$ a characteristic pressure value; $K_{s}$ the saturated hydraulic conductivity; s the fractal dimension relative to the Euclidean dimension; and m and n are shape parameters of the curve, dimensionless and positive.

Relative fractal dimension, s=D/E, expressed as the ratio of the soil fractal dimension (D) and the Euclidean dimension of the physical space (E) is related to the soil total volumetric porosity $\left(\phi \right)$, is defined by:

(6) $${\left(1-\phi \right)}^{s}+\phi^{2s}=1$$

### 4.2. Transport Model

The gentamicin transport in the soil, which essentially follows the movement of water in the soil, is studied with the advection–dispersion equation, which in its one-dimensional form is written as [21]:

(7) $$\frac{\partial \left(\theta C\right)}{\partial t}+\frac{\partial q_{s}}{\partial z}={ϒ}_{s}$$

considering that $q_{s}$ is governed by dynamic law:

(8) $$q_{s}=qC-\theta Da\frac{\partial C}{\partial z}$$

where C is the concentration of the antibiotic in the soil; q is the Darcy water flow velocity; Da is the diffusion coefficient of the antibiotic in water; and ${ϒ}_{s}$ is a sink term where antibiotic gains or losses due to chemical reactions are included. The variables q and θ are obtained from the water flow model.

Generally, in studies of small time scales such as the shallow infiltration process in a porous medium, the gas phase is not considered for water transfer modeling purposes, and since there are no chemical reactions, the term ${ϒ}_{s}$ is equal to zero [74,75]. However, the substance is also adsorbed on the solid phase, which is why it is necessary to know the ratio between the substance that transports the water and the substance that adsorbs and exchanges in the soil solid phase; this ratio is known as the adsorption isotherm [21].

Adsorption isotherms can have different shapes and depend mainly on the characteristics of the adsorbent, the adsorption surface, and sometimes on other constituents in the solution [21]. There are three types of adsorption isotherms: Freundlich, Langmuir, and linear. The latter is the one used in this work:

(9) $$\bar{C}=K_{d}C$$

where $\bar{C}$ is the amount of antibiotic retained by the soil (mg/kg); C is the concentration of antibiotic present in the equilibrium solution (mg/L); and $K_{d}$ is the distribution coefficient in the linear model (L/kg).

### 4.3. Characteristics of the Gentamicin Used in the Experiment

Gentamicin is an aminoglycoside antibiotic widely used in both scientific research and clinical applications. In its analytical or research-grade form, gentamicin is provided as a sterile, endotoxin-free solution, 0.1 μm filtered, with a standard concentration of 50 mg/mL in deionized water. The formulation used in this study is specifically designed for applications in cell culture and biotechnology, where precise concentration and the absence of contaminants are critical for experiment reproducibility and result validation [76]. Its main applications include antimicrobial sensitivity testing, toxicity assays, bacterial contamination control in cultures, and in vitro pharmacokinetic studies.

In contrast, gentamicin sulfate for clinical and pharmaceutical use is formulated as a stabilized salt for parenteral administration. The commercial presentation used in this research (gentamicin injectable solution 160 mg/mL from Laboratorios AMSA, Mexico City, Mexico) contains gentamicin sulfate at a concentration equivalent to 80 mg/mL, intended for intramuscular or intravenous administration under medical prescription. This pharmaceutical form complies with current pharmacopeial specifications and is intended for the treatment of severe bacterial infections in humans, where chemical stability, bioavailability, and clinical safety are essential [77].

The relationship between these two products lies in their common active ingredient: gentamicin base. While analytical-grade gentamicin is used in laboratory contexts with certified purity for research purposes, gentamicin sulfate represents its salt form, necessary for the formulation of stable and effective medications. The key difference between the two lies in the effective concentration of gentamicin base relative to the total compound weight: in gentamicin sulfate, the declared amount refers to the weight of the salt, not solely the active antibiotic. This implies that conversions for therapeutic dosing must account for this equivalence. Moreover, the conditions of use, applicable regulations, and purity grades differ significantly, with analytical gentamicin classified as a laboratory reagent and gentamicin sulfate as a regulated clinical drug [78].

Different input concentrations were applied to the soil: analytical-grade gentamicin was used at 150 mg/L, while gentamicin sulfate was applied at 300 mg/L. This methodological approach is based on assessing soil vulnerability to antimicrobial contaminants, particularly in ecotoxicology and bioremediation studies [63]. Soil sensitivity to gentamicin depends on variables such as texture, organic matter content, pH, and microbial activity. Therefore, using differentiated concentrations allows for the modeling of both realistic and high-load exposure scenarios [79].

### 4.4. Experiment

**Figure 5:**
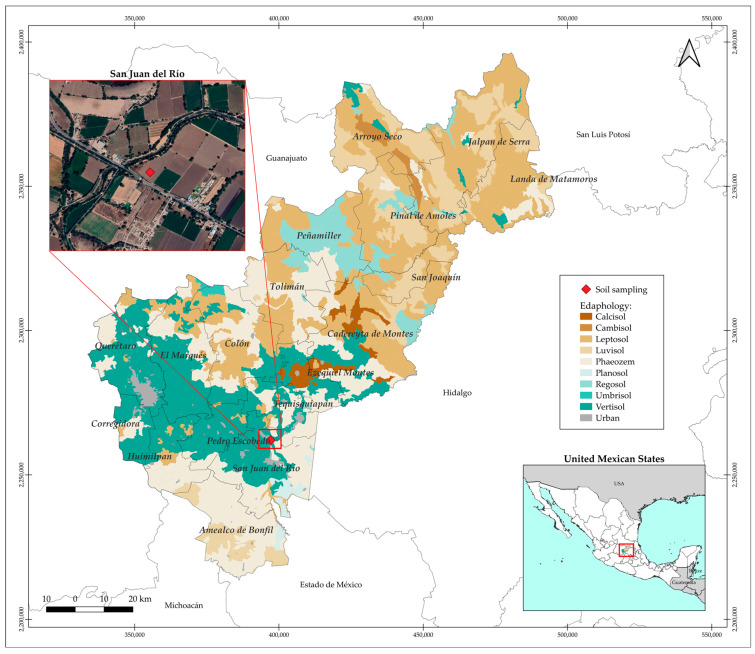
Geographic location of the soil sampling site within Irrigation District 023, San Juan del Río, Querétaro, Mexico.

A sample was collected from an agricultural plot within the Irrigation District 023, San Juan del Rio Queretaro, Mexico (Figure 5). It was sieved using No. 10 mesh (2 mm) to eliminate gravels and dried for a period of 10 days in the open air. Bulk density at the sampling site was performed using the known volume cylinder method and the organic matter content of the soil samples was determined in the laboratory using gravimetric analysis (loss-on-ignition method), while soil pH was measured by potentiometry, in accordance with the procedures established by the Mexican Official Standard NOM-021-SEMARNAT-2000 [80].

**Figure 6:**
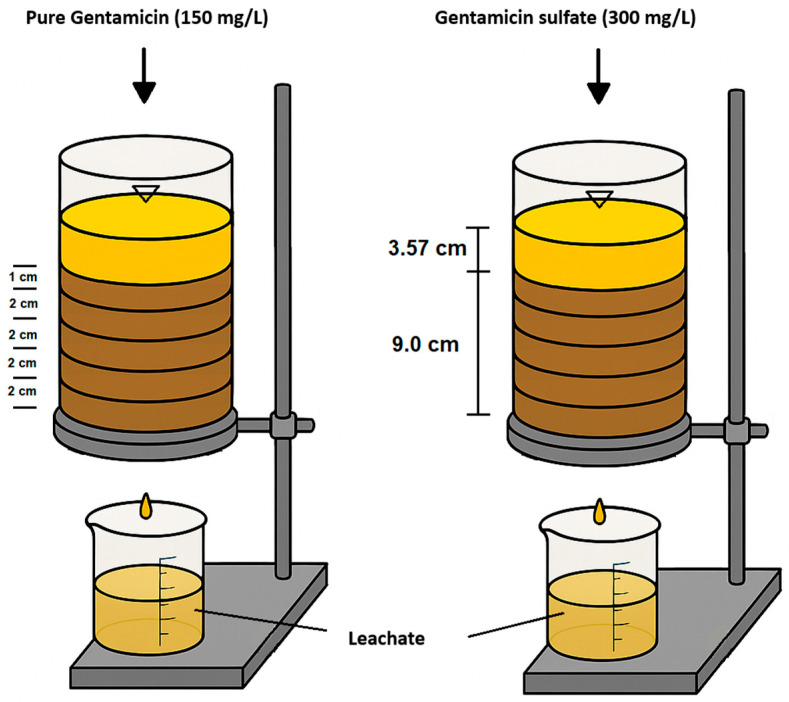
Experimental setup used for the infiltration tests. The left column corresponds to pure gentamicin solution (150 mg/L), and the right to gentamicin sulfate solution (300 mg/L).

The infiltration tests were conducted in transparent acrylic columns of 12.7 cm in diameter and 14 cm in length, which were cut into four sections of 2 cm in height on a CNC lathe with the support of a vernier. A porous layer covered with filter paper was placed at the base of the column to retain the soil and favor the exit of water and air during the infiltration process. In addition, a funnel was placed to convey the leachate to the containers (Figure 6).

Before the soil was placed in the acrylic columns, the walls of the tube were covered with a thin layer of kerosene to prevent preferential flow. In addition, 3 samples were taken and sent to the laboratory to obtain the initial moisture content using the gravimetric method. The soil was placed inside the column in 2 cm blocks with a bulk density similar to that obtained at the place where the samples were taken.

In the laboratory, the granulometric curve was obtained by mesh analysis following the methodology proposed by the United States Department of Agriculture (USDA) and the texture with the Bouyoucos hydrometer in accordance with the NOM-021-SEMARNAT-2000 standard. In addition, pH, organic matter content (%), and total organic carbon (%) were measured.

### 4.5. Determination of Gentamicin Concentration in Soil

Gentamicin concentrations in the soil were analyzed using spectrophotometric techniques at the Research Center in Chemistry for the Circular Economy (CIQEC), affiliated with the Autonomous University of Queretaro, Mexico. The following procedures were carried out:

Gentamicin was extracted from the soil following the method recommended by [81]. Specifically, 5 g of each soil sample was dissolved in 40 mL of a 1:1 mixture of methanol and distilled water. The solution was then subjected to an ultrasonic bath at 40 kHz for 30 min, followed by centrifugation at 6000 rpm for 15 min. The resulting supernatant was filtered through a 0.45 µm membrane filter. The filtrate was used to determine gentamicin concentrations in the soil, following the methods implemented by [82,83,84], in which absorbance was measured at 193 nm using a spectrophotometer. The gentamicin concentration was then determined according to the following expression:

(10) $$\text{Concentration}=\frac{\text{absorbance}-0.0992}{0.0009}$$

The regression model yielded a coefficient of determination (R^2^) of 0.9822.

## 5. Conclusions

The use of numerical models to solve the differential equations governing water transfer and chemical transport in soil has gained increasing relevance in recent years. In addition to their flexibility in handling changes in boundary conditions, the exponential growth in computing power has made them increasingly widely used.

This made it possible to simulate two scenarios involving the two types of gentamicin used: soil flushing after antibiotic discharge and continued application of the same concentration over a longer period. In both cases, gentamicin sulfate exhibited greater mobility in the soil compared to pure gentamicin, primarily due to its high solubility in water. Although the same type of soil was used in both scenarios, it was observed that gentamicin sulfate increased the water infiltration rate by a factor of 1.55 compared to pure gentamicin.

Finally, the model presented in this study is proposed as a tool for decision makers to consider the potential harm caused by the disposal of antibiotics into drainage systems. Since such waste can come into contact with soil at some point along its pathway, the repercussions are not limited to increased microbial resistance due to the use of manure and sludge as fertilizers in crops. They also include the continuous introduction, even at low concentrations, of these compounds into water bodies. This may have significant impacts on irrigated crops, animals that consume contaminated water, aquatic ecosystems, and ultimately on human populations that rely on these water sources.

## Figures and Tables

**Table 1:** Measured and estimated physical and hydraulic parameters of the soil samples. The suffixes (c1) and (c2) indicate values corresponding to the tests with pure gentamicin and gentamicin sulfate, respectively.

Parameter	Value	Parameter	Value
Sand $\left(\%\right)$	15.23	s	0.7019
Clay $\left(\%\right)$	61.43	$RMSE_{GC}$	0.0166
Texture	23.34	h $\left(cm\right)$	3.5
L $\left(cm\right)$	8	$K_{s}(c1)$ $\left(cm/h\right)$	0.9481
$\rho_{a}$ $\left(g/cm^{3}\right)$	1.35	$\Psi_{d}(c1)$ $\left(-cm\right)$	92.8674
$\theta_{s}$ $\left(cm^{3}/cm^{3}\right)$	0.4906	$RMSE_{IC}(c1)$	0.3084
$D_{d}$ $\left(\mu m\right)$	1136.6942	$K_{s}(c2)$ $\left(cm/h\right)$	1.4689
m	0.18609	$\Psi_{d}(c2)$ $\left(-cm\right)$	47.0628
n	3.1784	$RMSE_{IC}(c2)$	0.2170

**Table 2:** Measured and calculated chemical properties of the soil samples.

Parameter	Value
pH	6.05
Organic matter (%)	2.61
Total organic carbon (%)	1.58

**Table 3:** Dispersion coefficient values and root mean square error (RMSE) for the fitted advection–dispersion model using pure gentamicin and gentamicin sulfate.

Parameter	Pure Gentamicin	Gentamicin Sulfate
Dispersion coefficient D (m^2^/s)	9.55 × 10^−3^	2.97 × 10^−2^
RMSE	0.2257	0.1889

## Data Availability

The original contributions presented in this study are included in the article. Further inquiries can be directed to the corresponding authors.

## References

[1] Van Boeckel T.P., Brower C., Gilbert M., Grenfell B.T., Levin S.A., Robinson T.P., Teillant A., Laxminarayan R. (2015). Global Trends in Antimicrobial Use in Food Animals. Proc. Natl. Acad. Sci. USA.

[2] Gogoi A., Mazumder P., Tyagi V.K., Tushara Chaminda G.G., An A.K., Kumar M. (2018). Occurrence and Fate of Emerging Contaminants in Water Environment: A Review. Groundw. Sustain. Dev..

[3] Massey L.B., Haggard B.E., Galloway J.M., Loftin K.A., Meyer M.T., Green W.R. (2010). Antibiotic Fate and Transport in Three Effluent-Dominated Ozark Streams. Ecol. Eng..

[4] Li S., Shi W., Li H., Xu N., Zhang R., Chen X., Sun W., Wen D., He S., Pan J. (2018). Antibiotics in Water and Sediments of Rivers and Coastal Area of Zhuhai City, Pearl River Estuary, South China. Sci. Total Environ..

[5] Nemergut D.R., Schmidt S.K., Fukami T., O’Neill S.P., Bilinski T.M., Stanish L.F., Knelman J.E., Darcy J.L., Lynch R.C., Wickey P. (2013). Patterns and Processes of Microbial Community Assembly. Microbiol. Mol. Biol. Rev..

[6] Wang X., Chi Y., Song S. (2024). Important Soil Microbiota’s Effects on Plants and Soils: A Comprehensive 30-Year Systematic Literature Review. Front. Microbiol..

[7] Pepper I.L., Gerba C.P., Gentry T.J. (2015). Environmental Microbiology.

[8] Bayranvand M., Akbarinia M., Salehi Jouzani G., Gharechahi J., Kooch Y., Baldrian P. (2021). Composition of Soil Bacterial and Fungal Communities in Relation to Vegetation Composition and Soil Characteristics along an Altitudinal Gradient. FEMS Microbiol. Ecol..

[9] Batuman O., Britt-Ugartemendia K., Kunwar S., Yilmaz S., Fessler L., Redondo A., Chumachenko K., Chakravarty S., Wade T. (2024). The Use and Impact of Antibiotics in Plant Agriculture: A Review. Phytopathology.

[10] Coates J., Bostick K.J., Jones B.A., Caston N., Ayalew M. (2022). What Is the Impact of Aminoglycoside Exposure on Soil and Plant Root-Associated Microbiota? A Systematic Review Protocol. Environ. Evid..

[11] Sanchez-Cid C., Guironnet A., Wiest L., Vulliet E., Vogel T.M. (2021). Gentamicin Adsorption onto Soil Particles Prevents Overall Short-Term Effects on the Soil Microbiome and Resistome. Antibiotics.

[12] Cycoń M., Mrozik A., Piotrowska-Seget Z. (2019). Antibiotics in the Soil Environment—Degradation and Their Impact on Microbial Activity and Diversity. Front. Microbiol..

[13] Le-Minh N., Khan S.J., Drewes J.E., Stuetz R.M. (2010). Fate of Antibiotics during Municipal Water Recycling Treatment Processes. Water Res..

[14] Sanchez-Cid C., Guironnet A., Keuschnig C., Wiest L., Vulliet E., Vogel T.M. (2022). Gentamicin at Sub-Inhibitory Concentrations Selects for Antibiotic Resistance in the Environment. ISME Commun..

[15] Bassil R.J., Bashour I.I., Sleiman F.T., Abou-Jawdeh Y.A. (2013). Antibiotic Uptake by Plants from Manure-Amended Soils. J. Environ. Sci. Health B.

[16] Miller S.A., Ferreira J.P., LeJeune J.T. (2022). Antimicrobial Use and Resistance in Plant Agriculture: A One Health Perspective. Agriculture.

[17] Kushner B., Allen P.D., Crane B.T. (2016). Frequency and Demographics of Gentamicin Use. Otol. Neurotol..

[18] National Center for Biotechnology Information PubChem Compound Summary for Gentamicin Sulfate. https://pubchem.ncbi.nlm.nih.gov/compound/Gentamicin-Sulfate.

[19] Barrios R.E., Bartelt-Hunt S.L., Li Y., Li X. (2021). Modeling the Vertical Transport of Antibiotic Resistance Genes in Agricultural Soils Following Manure Application. Environ. Pollut..

[20] Tong X., Mohapatra S., Zhang J., Tran N.H., You L., He Y., Gin K.Y.-H. (2022). Source, Fate, Transport and Modelling of Selected Emerging Contaminants in the Aquatic Environment: Current Status and Future Perspectives. Water Res..

[21] Zheng C., Bennett G.D. (2002). Applied Contaminant Transport Modeling.

[22] Jamal M.S., Awotunde A.A., Al-Kobaisi M.S., Al-Yousef H.Y., Sadeed A., Patil S. (2023). Interwell Simulation Model for the Advection Dispersion Equation. Comput. Geosci..

[23] Fuentes S., Trejo-Alonso J., Quevedo A., Fuentes C., Chávez C. (2020). Modeling Soil Water Redistribution under Gravity Irrigation with the Richards Equation. Mathematics.

[24] Chen J.-S., Chen J.-T., Liu C.-W., Liang C.-P., Lin C.-W. (2011). Analytical Solutions to Two-Dimensional Advection–Dispersion Equation in Cylindrical Coordinates in Finite Domain Subject to First- and Third-Type Inlet Boundary Conditions. J. Hydrol..

[25] Moranda A., Cianci R., Paladino O. (2018). Analytical Solutions of One-Dimensional Contaminant Transport in Soils with Source Production-Decay. Soil Syst..

[26] Fuentes S., Chávez C., Brambila-Paz F., Trejo-Alonso J. (2022). Hydrodynamic Border Irrigation Model: Comparison of Infiltration Equations. Water.

[27] Morales-Durán N., Fuentes S., Chávez C. (2023). A Soil Database from Queretaro, Mexico for Assessment of Crop and Irrigation Water Requirements. Sci. Data.

[28] Richards L.A. (1931). Capillary Conduction of Liquids through Porous Mediums. Physics.

[29] Šimůnek J., Van Genuchten M.T., Šejna M. (2006). The HYDRUS Software Package for Simulating Two-and Three-Dimensional Movement of Water, Heat, and Multiple Solutes in Variably Saturated Media. Tech. Man. Version.

[30] Šimůnek J., Van Genuchten M.T., Šejna M. (2016). Recent Developments and Applications of the HYDRUS Computer Software Packages. Vadose Zone J..

[31] Mertz S., Devau N., Thouin H., Battaglia-Brunet F., Norini M.-P., Crampon M., Le Forestier L. (2025). Leaching of Pollutant Metals (Pb, Zn) from Abandoned Mine Tailings: A Multicomponent Reactive Transport Model of a Pilot-Scale Experiment. Sci. Total Environ..

[32] Kamil H., Soulaïmani A., Beljadid A. (2025). A Comparative Study of Physics-Informed Neural Network Strategies for Modeling Water and Nitrogen Transport in Unsaturated Soils. J. Hydrol..

[33] Turkeltaub T., Jia X., Zhu Y., Shao M., Binley A. (2021). A Comparative Study of Conceptual Model Complexity to Describe Water Flow and Nitrate Transport in Deep Unsaturated Loess. Water Resour. Res..

[34] Wehbe S., Zewge F., Inagaki Y., Sievert W., Nutakki T.U.K., Deshpande A. (2023). A Mechanistic Model for Simulation of Carbendazim and Chlorothalonil Transport through a Two-Stage Vertical Flow Constructed Wetland. Water.

[35] Fuentes C., Brambila-Paz F., Haverkamp R. (2017). Soil Hydrodynamic Characteristics. Gravity Irrigation.

[36] Moré J.J. (1978). The Levenberg-Marquardt Algorithm: Implementation and Theory. Numerical Analysis.

[37] Fuentes S., Fuentes C., Chávez C. (2022). Parameter Optimization of Green and Ampt Equation Using a Nonlinear Algorithm. INAGBI.

[38] Haverkamp R., Leij F.J., Fuentes C., Sciortino A., Ross P.J. (2005). Soil Water Retention: I. Introduction of a Shape Index. Soil Sci. Soc. Am. J..

[39] Samreen, Ahmad I., Malak H.A., Abulreesh H.H. (2021). Environmental Antimicrobial Resistance and Its Drivers: A Potential Threat to Public Health. J. Glob. Antimicrob. Resist..

[40] Sambaza S.S., Naicker N. (2023). Contribution of Wastewater to Antimicrobial Resistance: A Review Article. J. Glob. Antimicrob. Resist..

[41] Wang F., Fu Y.-H., Sheng H.-J., Topp E., Jiang X., Zhu Y.-G., Tiedje J.M. (2021). Antibiotic Resistance in the Soil Ecosystem: A One Health Perspective. Curr. Opin. Environ. Sci. Health.

[42] Fernández Rodríguez R.E. (2021). Antibiotic resistance: The role of man, animals and the environment. Sun.

[43] Forsberg K.J., Reyes A., Wang B., Selleck E.M., Sommer M.O.A., Dantas G. (2012). The Shared Antibiotic Resistome of Soil Bacteria and Human Pathogens. Science.

[44] Han B., Ma L., Yu Q., Yang J., Su W., Hilal M.G., Li X., Zhang S., Li H. (2022). The Source, Fate and Prospect of Antibiotic Resistance Genes in Soil: A Review. Front. Microbiol..

[45] Telo da Gama J. (2023). The Role of Soils in Sustainability, Climate Change, and Ecosystem Services: Challenges and Opportunities. Ecologies.

[46] Ahmed S.K., Hussein S., Qurbani K., Ibrahim R.H., Fareeq A., Mahmood K.A., Mohamed M.G. (2024). Antimicrobial Resistance: Impacts, Challenges, and Future Prospects. J. Med. Surg. Public Health.

[47] Reygaert W.C. (2018). An Overview of the Antimicrobial Resistance Mechanisms of Bacteria. AIMS Microbiol..

[48] Guo K., Han L., Luo J., Lu G., Li Y., Liu J. (2025). Occurrence and Accumulation Characteristics of Antibiotics in Soil and Effects of Carbon and Nitrogen Cycle. Curr. Opin. Environ. Sci. Health.

[49] Jia W.-L., Song C., He L.-Y., Wang B., Gao F.-Z., Zhang M., Ying G.-G. (2023). Antibiotics in Soil and Water: Occurrence, Fate, and Risk. Curr. Opin. Environ. Sci. Health.

[50] Chow L.K.M., Ghaly T.M., Gillings M.R. (2021). A Survey of Sub-Inhibitory Concentrations of Antibiotics in the Environment. J. Environ. Sci..

[51] Grenni P., Ancona V., Barra Caracciolo A. (2018). Ecological Effects of Antibiotics on Natural Ecosystems: A Review. Microchem. J..

[52] Li Y., Li R., Hou J., Sun X., Wang Y., Li L., Yang F., Yao Y., An Y. (2024). Mobile Genetic Elements Affect the Dissemination of Antibiotic Resistance Genes (ARGs) of Clinical Importance in the Environment. Environ. Res..

[53] Westhoff S., van Leeuwe T.M., Qachach O., Zhang Z., van Wezel G.P., Rozen D.E. (2017). The Evolution of No-Cost Resistance at Sub-MIC Concentrations of Streptomycin in Streptomyces Coelicolor. ISME J..

[54] Parras-Moltó M., Lund D., Ebmeyer S., Larsson D.G.J., Johnning A., Kristiansson E. (2025). The Transfer of Antibiotic Resistance Genes between Evolutionarily Distant Bacteria. mSphere.

[55] Zhou Y., Niu L., Zhu S., Lu H., Liu W. (2017). Occurrence, Abundance, and Distribution of Sulfonamide and Tetracycline Resistance Genes in Agricultural Soils across China. Sci. Total Environ..

[56] Vydrin A.F., Shikhaleev I.V., Makhortov V.L., Shcherenko N.N., Kolchanova N.V. (2003). Component Composition of Gentamicin Sulfate Preparations. Pharm. Chem. J..

[57] Doucette W.J., Andren A.W. (1988). Aqueous Solubility of Selected Biphenyl, Furan, and Dioxin Congeners. Chemosphere.

[58] Puzyn T., Mostrąg A., Falandysz J., Kholod Y., Leszczynski J. (2009). Predicting Water Solubility of Congeners: Chloronaphthalenes—A Case Study. J. Hazard. Mater..

[59] Zuluaga A.F., Agudelo M., Cardeño J.J., Rodriguez C.A., Vesga O. (2010). Determination of Therapeutic Equivalence of Generic Products of Gentamicin in the Neutropenic Mouse Thigh Infection Model. PLoS ONE.

[60] Khaled E., Khalil M.M., Abed El Aziz G.M. (2017). Calixarene/Carbon Nanotubes Based Screen Printed Sensors for Potentiometric Determination of Gentamicin Sulphate in Pharmaceutical Preparations and Spiked Surface Water Samples. Sens. Actuators B Chem..

[61] Pisani S., Piazza A., Dorati R., Genta I., Rosalia M., Chiesa E., Bruni G., Migliavacca R., Conti B. (2025). Investigating Electrospun Shape Memory Patches as Gentamicin Drug Delivery System. Int. J. Pharm..

[62] Sandaoui M., Aboulfadile M.A., Sakoui S., Derdak R., El Khalfi B., El Ghachtouli S., Azzi M., Zaroual Z. (2024). The Ultrasonic Degradation of a Pharmaceutical Formulation Including Gentamicin Sulfate and Parabens: Optimization of Operational Parameters, Antibacterial Activity Assessment, and Analysis of Resulting by-Products. J. Water Process Eng..

[63] Wang D., Zhang Q., Sun Z., Li Y., Xie X., Xin S., Liu G., Liu H., Xin Y. (2025). Decoding the Fate of Antibiotic Resistance Genes in Soil with the Application of Thermally Treated Gentamicin Mycelial Residues. Bioresour. Technol. Rep..

[64] Liu Y., Cheng D., Xue J., Feng Y., Wakelin S.A., Weaver L., Shehata E., Li Z. (2022). Fate of Bacterial Community, Antibiotic Resistance Genes and Gentamicin Residues in Soil after Three-Year Amendment Using Gentamicin Fermentation Waste. Chemosphere.

[65] Arshad M.S., Zafar S., Rana S.J., Nazari K., Chang M.-W., Ahmad Z. (2023). Fabrication of Gentamicin Sulphate Laden Stimulus Responsive Polymeric Microarray Patches for the Treatment of Bacterial Biofilms. J. Drug Deliv. Sci. Technol..

[66] Koestel J.K., Moeys J., Jarvis N.J. (2012). Meta-Analysis of the Effects of Soil Properties, Site Factors and Experimental Conditions on Solute Transport. Hydrol. Earth Syst. Sci..

[67] Mojid M.A., Hossain A.B.M.Z., Wyseure G.C.L. (2018). Relation of Reactive Solute-Transport Parameters to Basic Soil Properties. Eurasian J. Soil Sci..

[68] Merck KGaA (Sigma-Aldrich) Gentamicin Solution—Product Information: 50 Mg/mL, BioReagent, Suitable for Cell Culture. https://www.sigmaaldrich.com/MX/es/product/sigma/g1397.

[69] Tabuada M.A., Rego Z.J.C., Vachaud G., Pereira L.S. (1995). Two-Dimensional Infiltration under Furrow Irrigation: Modelling, Its Validation and Applications. Agric. Water Manag..

[70] Castanedo V., Saucedo H., Fuentes C. (2019). Modeling Two-Dimensional Infiltration with Constant and Time-Variable Water Depth. Water.

[71] Darcy H. (1856). Les Fontaines Publiques de La Ville de Dijon.

[72] van Genuchten M.T. (1980). A Closed-Form Equation for Predicting the Hydraulic Conductivity of Unsaturated Soils. Soil Sci. Soc. Am. J..

[73] Fuentes C., Chávez C., Brambila F. (2020). Relating Hydraulic Conductivity Curve to Soil-Water Retention Curve Using a Fractal Model. Mathematics.

[74] Zerihun D., Furman A., Warrick A.W., Sanchez C.A. (2005). Coupled Surface-Subsurface Flow Model for Improved Basin Irrigation Management. J. Irrig. Drain. Eng..

[75] Chávez C., Fuentes C., Brambila-Paz F., Castañeda A. (2014). Numerical Solution of the Advection-Dispersion Equation: Application to the Agricultural Drainage. J. Agr. Sci. Tech..

[76] Sigma-Aldrich Protocol Guide: WST-1 Assay for Cell Proliferation and Viability.

[77] AMSA Laboratorios (2025). Gentamicina Solución Inyectable 160 Mg/2 mL: Información Técnica del Producto.

[78] World Health Organization (2024). WHO Model List of Essential Medicines—2023 Update.

[79] Kim S.H., Park S.Y., Kim G.E., Jho E.H. (2023). Effect of pH and Temperature on the Biodegradation of Oxytetracycline, Streptomycin, and Validamycin A in Soil. Appl. Biol. Chem..

[80] SEMARNAT (2002). Norma Oficial Mexicana NOM-021-SEMARNAT-2000 Que Establece las Especificaciones de Fertilidad, Salinidad y Clasificación de Suelos. Estudios, Muestreo y Análisis [Official Mexican Standard NOM-021-SEMARNAT-2000: Specifications of Fertility, Salinity and Soil Classification. Studies, Sampling and Analysis].

[81] Rashtbari M., Safari Sinegani A.A. (2020). Efficiency of Soil Extracellular Enzymes in Soils Treated by Organic and Mineral Conditioners Against Mostly Applied Veterinary Antibiotics (Gentamicin, Oxytetracycline and Penicillin). J. Soil Manag. Sustain. Prod..

[82] Naveed S., Shah S.N., Qamar F., Waheed N., Nazeer S. (2014). Simple UV Spectrophotometric Assay of New Formulation Gentamycin. J. App. Pharm..

[83] Pareja-Aivar L. (2012). Desarrollo y Validación de Un Método Análitico Para La Cuantificación de Gentamicina En Un Producto Farmacéutico En Crema Por Potencia Antibiotica.

[84] Hernández-García R.G. (2019). Determinación de La Cinética de Liberación de Gentamicina En Cemento Óseo Afectado Por El Desgaste y La Influencia de La Materia Orgánica.

